# Development and validation of a nomogram for predicting immune-mediated colitis in lung cancer patients treated with immune checkpoint inhibitors: a retrospective cohort study in China

**DOI:** 10.3389/fimmu.2025.1510053

**Published:** 2025-01-30

**Authors:** Qianjie Xu, Xiaosheng Li, Yuliang Yuan, Guangzhong Liang, Zuhai Hu, Wei Zhang, Ying Wang, Haike Lei

**Affiliations:** ^1^ Chongqing Cancer Multi-omics Big Data Application Engineering Research Center, Chongqing University Cancer Hospital, Chongqing, China; ^2^ Chongqing Key Laboratory of Translational Research for Cancer Metastasis and Individualized Treatment, Chongqing University Cancer Hospital, Chongqing, China; ^3^ Department of Health Statistics, School of Public Health, Chongqing Medical University, Chongqing, China

**Keywords:** ICIS, nomogram, risk factors, prediction model, IMC

## Abstract

**Background:**

The increasing utilization of immune checkpoint inhibitors (ICIs) has led to a concomitant rise in the incidence of immune-related adverse events (irAEs), notably immune-mediated colitis (IMC). This study aimed to identify the clinical risk factors associated with IMC development in patients with lung cancer and to develop a risk prediction model to facilitate personalized treatment and care strategies.

**Methods:**

The data collected included 21 variables, including sociodemographic characteristics, cancer-related factors, and routine blood markers. The dataset was randomly partitioned into a training set (70%) and a validation set (30%). Univariate and multivariate logistic regression analyses were conducted to identify independent predictors of IMC development. On the basis of the results of the multivariate analysis, a nomogram prediction model was developed. Model performance was assessed via the area under the receiver operating characteristic curve (AUC), calibration curve analysis, decision curve analysis (DCA), and clinical impact curve (CIC).

**Results:**

Among the 2103 patients, 66 (3.14%) developed IMCs. Multivariate logistic regression analysis revealed female sex, small cell lung cancer (SCLC), elevated β2 microglobulin (β2-MG) and globulin (GLB) levels, and an increased neutrophil−lymphocyte ratio (NLR) as independent predictors of IMC development (all *P* < 0.05). Conversely, a higher white blood cell (WBC) count, CD4/CD8 ratio, and platelet−lymphocyte ratio (PLR) were identified as factors associated with a reduced risk of IMC development (all *P* < 0.05). The nomogram prediction model demonstrated good discrimination, achieving an AUC of 0.830 (95% CI: 0.774–0.887) in the training set and 0.827 (95% CI: 0.709–0.944) in the validation set. Analysis of the calibration curve, DCA, and CIC indicated good predictive accuracy and clinical utility of the developed model.

**Conclusion:**

This study identified eight independent predictors of IMC development in patients with lung cancer and subsequently developed a nomogram-based prediction model to assess IMC risk. Utilization of this model has the potential to assist clinicians in implementing appropriate preventive and therapeutic strategies, ultimately contributing to a reduction in the incidence of IMC among this patient population.

## Introduction

Lung cancer remains a leading cause of cancer-related mortality worldwide, with China experiencing persistently high incidence and mortality rates (1). Over the past few decades, immune checkpoint inhibitors (ICIs), which are antibodies that target inhibitory immune checkpoint molecules such as programmed cell death-1 (PD-1), programmed cell death ligand-1 (PD-L1), and cytotoxic T-lymphocyte antigen-4 (CTLA-4), have revolutionized cancer treatment. ICI therapies have demonstrated substantial clinical benefits, significantly improving overall survival in patients with advanced lung cancer and transforming the standard of care (2, 3). ICIs have largely replaced or are used in combination with conventional treatment modalities such as chemotherapy and radiotherapy. However, the expanding utilization of ICIs has led to a concomitant increase in the reported incidence of immune-related adverse events (irAEs) (4). IrAEs can manifest in any organ system, with the skin and gastrointestinal tract being among the most frequently affected sites. Among these irAEs, immune-mediated colitis (IMC) is highly prevalent (5).

Accurately determining the precise incidence and prevalence of IMCs is challenging, as these values vary considerably depending on the specific ICI treatment regimen and individual patient characteristics. Specifically, the highest incidence rates are reported in patients receiving CTLA-4 inhibitor monotherapy (3.4–15.5%), followed by those receiving combination therapy with CTLA-4 and PD-1 inhibitors (0.7–12.8%). The lowest incidence is observed in patients treated with PD-1/PD-L1 inhibitors (0.7–2.6%) (6). IMC onset can occur as early as one week after initial ICI administration, with a median time to onset of 4–8 weeks (7). Characterized by colonic mucosal inflammation, IMC presents with a heterogeneous spectrum of nonspecific clinical manifestations, including diarrhea (92%), abdominal pain (82%), hematochezia (64%), fever (46%), and vomiting (8). The severity of IMC is highly variable, ranging from mild diarrhea to severe complications such as bowel perforation and even mortality. Notably, a meta-analysis reported a 5% overall mortality rate associated with IMC (9). The heterogeneous clinical presentation and potential for rapid disease progression underscore the critical importance of early detection and timely intervention in cases of IMC.

Traditionally, a presumptive diagnosis of IMC is considered when a patient receiving ICI therapy experiences diarrhea and abdominal pain, supported by endoscopic and/or histopathological findings from gastrointestinal biopsies (10). However, the clinical, endoscopic, and histological features of IMC are nonspecific. Consequently, the traditional diagnostic approach for IMC relies on a process of elimination, requiring the exclusion of all other competing etiologies, including infectious colitis, drug-induced colitis, and graft-versus-host disease (GvHD) (11). This lengthy process can delay diagnosis, potentially exacerbating patient symptoms.

Therefore, a clinical model capable of rapidly identifying patients at risk of developing IMC is urgently needed. Nomograms are powerful graphical tools or algorithms that integrate biological and clinical variables, demonstrating wide applicability in oncology for risk factor identification and risk prediction (12). For example, Tong et al. utilized a nomogram model to investigate the incidence and predictive factors of prolonged chest tube drainage after video-assisted thoracoscopic surgery for the rapid prediction of this complication in clinical practice (13).

This study aims to leverage a nomogram algorithm to identify risk factors associated with IMC development in patients with lung cancer and to develop a robust risk prediction model. Ultimately, this model has the potential to complement traditional diagnostic approaches by enabling rapid risk stratification and informing personalized treatment and care decisions.

## Materials and methods

### Study population and inclusion and exclusion criteria

This retrospective study involved a comprehensive analysis of data obtained from 2290 patients diagnosed with lung cancer who received ICI therapy at the hospital between January 1, 2020, and May 31, 2024. Data regarding patient demographics, clinical characteristics, and laboratory results were extracted from electronic medical records. The following data were collected: sociodemographic characteristics, age, sex, body mass index (BMI), history of hypertension, and diabetes status. Tumor-related data: Tumor type and stage. Hematological parameters: White blood cell count (WBC), hemoglobin (Hb), lactate dehydrogenase (LDH), β2 microglobulin (β2-MG), calcium (Ca), B-cell count, T-cell count, natural killer (NK) count, albumin (ALB), globulin (GLB), the CD4/CD8 ratio, the platelet−lymphocyte ratio (PLR), the neutrophil−lymphocyte ratio (NLR), and the lymphocyte−monocyte ratio (LMR). The diagnosis of IMC was established on the basis of the presence of diarrhea and abdominal pain following ICI initiation, in conjunction with positive endoscopic and/or histopathological findings from gastrointestinal biopsies, after excluding other potential etiologies of colitis. All blood tests were performed at the accredited laboratory of the Chongqing university cancer hospital.

**Figure 1:**
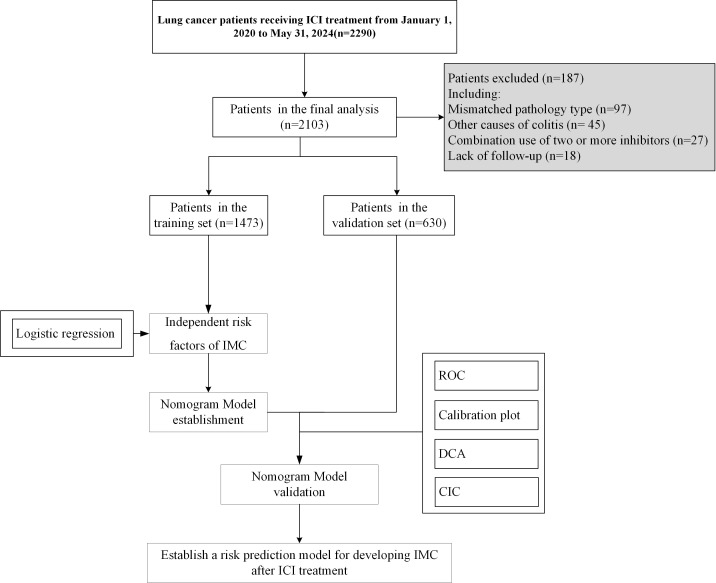
Flow chart of the patients enrolled in the final study cohorts.

Patients were eligible for inclusion if they met the following criteria: (1) aged ≥ 18 years; (2) had at least one hospitalization; and (3) received ICI therapy with any of three inhibitors: CTLA-4, PD-1, or PD-L1. Patients were excluded from the study if they met any of the following criteria: (1) missing data for critical variables, including tumor stage or hematological parameters; (2) death within 48 hours of hospital admission; (3) colitis attributed to other causes, such as infectious colitis, drug-induced colitis, or graft-versus-host disease (GvHD); or (4) concurrent use of two or more ICI agents. After passing the inclusion and exclusion criteria, 2103 patients were included in the model construction, as shown in **Figure 1**.

### Model construction and validation

Eligible patients were randomly assigned to either a training set (n=1473) or a validation set (n=630) at a ratio of 7:3. Randomization was performed via the “caret” package in R software with a fixed random seed to ensure reproducibility. In the training set, univariate logistic regression analyses were conducted to assess the associations between each clinical variable and the incidence of IMC. Variables with a *P* value < 0.2 in the univariate analysis were subsequently included in a stepwise multivariate logistic regression model to identify independent predictors of IMCs. On the basis of the results of the multivariate analysis, a nomogram prediction model was developed. The performance of the nomogram model was assessed in the validation set. The area under the receiver operating characteristic curve (AUC) was calculated using the “pROC” package to assess the generalizability of the Nomogram model (14). The “caret” package and “rms” package were used to perform 1000 bootstrap resamples to generate calibration curves, which were utilized to validate the predictive accuracy of the Nomogram model in both the training and validation datasets (15). Decision curve analysis (DCA) and clinical impact curves (CICs) were generated via the “rmda” package in R to evaluate the clinical utility and net benefit of the model across a range of threshold probabilities (16).

### Statistical analysis

Normally distributed continuous variables are expressed as the mean ± standard deviation (SD) and were compared via t tests. Nonnormally distributed continuous variables are presented as medians (M) and interquartile ranges (IQRs) and were compared via a Wilcoxon rank-sum test. Categorical variables are summarized as frequencies and percentages and were compared via the chi-square test or Fisher’s exact test, as appropriate. The above statistical methods were implemented using the “tableone” package in R (17). Missing data were imputed via the “mice” package in R (18). All the statistical analyses were performed via R version 4.1.2 (http://www.r-project.org). A two-tailed *P* value of < 0.05 was considered statistically significant.

## Results

### Clinical characteristics of the study population

After applying the inclusion and exclusion criteria, 2103 patients with lung cancer treated with ICIs were included in this study, of whom 66 (3.14%) developed IMCs. The mean age of the study population was 61.67 years, and 83.79% of the cohort was male. More than half of the patients (57.82%) had a BMI between 18.5 and 23.9. The majority of patients did not have comorbidities such as hypertension (77.46%) or diabetes (85.07%). Among the included patients, 78.89% had non-small cell lung cancer (NSCLC), and 96.67% presented with advanced-stage disease (stage III or IV). Statistically significant differences (all *P* < 0.05) were observed between patients who developed IMCs and those who did not, with respect to several hematological parameters, including WBC, Hb, β2-MG, T-cell count, B-cell count, GLB, the CD4/CD8 ratio, and the NLR. Specifically, patients who developed IMCs presented lower WBC counts, Hb levels, T-cell counts, B-cell counts, and CD4/CD8 ratios, along with higher β2-MG levels, GLB levels, and NLRs. The detailed situation is shown in **Table 1**.

**Table 1:** Sociodemographic and clinical characteristics of the patients.

Variable	Overall (n=2103)	No-IMC (n=2037)	IMC (n=66)	*P*
Age (Year)	61.67 ± 8.88	61.66 ± 8.86	62.00 ± 9.45	0.759
Gender (%)				0.001
Male	1762 (83.79)	1717 (97.45)	45 (2.55)	
Female	341 (16.21)	320 (93.84)	21 (6.16)	
BMI (%)				0.134
18.5-23.9	1216 (57.82)	1174 (96.55)	42 (3.45)	
≥24	750 (35.66)	733 (97.73)	17 (2.27)	
<18.5	137 (6.51)	130 (94.89)	7 (5.11)	
Hypertension (%)				0.852
NO	1629 (77.46)	1579 (96.93)	50 (3.07)	
YES	474 (22.54)	458 (96.62)	16 (3.38)	
Diabetes (%)				1.000
NO	1789 (85.07)	1733 (96.87)	56 (3.13)	
YES	314 (14.93)	304 (96.82)	10 (3.18)	
Pathology (%)				0.020
NSCLC	1659 (78.89)	1615 (97.35)	44 (2.65)	
SCLC	444 (21.11)	422 (95.05)	22 (4.95)	
Stage (%)				0.160
I-II	70 (3.33)	69 (98.57)	1 (1.43)	
III	565 (26.87)	553 (97.88)	12 (2.12)	
IV	1468 (69.81)	1415 (96.39)	53 (3.61)	
WBC (10^9^/L)	6.91 ± 2.89	6.96 ± 2.89	5.59 ± 2.46	<0.001
Hb (g/L)	124.40 ± 17.77	124.63 ± 17.61	117.14 ± 21.25	0.001
LDH (U/L) *	200.00 [171.00, 244.50]	200.00 [171.00, 244.00]	196.55 [172.50, 261.22]	0.710
β2-MG (mg/L)	2.85 ± 1.06	2.83 ± 1.01	3.59 ± 1.96	<0.001
Ca (mmol/L)	2.29 ± 0.15	2.29 ± 0.15	2.27 ± 0.16	0.339
T cell (%)	953.56 ± 418.84	957.45 ± 421.10	833.56 ± 321.77	0.018
B cell (%) *	107.00 [60.00, 178.00]	107.00 [61.00, 180.00]	79.00 [46.00, 145.00]	0.006
NK (%)	263.76 ± 177.55	264.03 ± 176.73	255.47 ± 202.61	0.700
ALB (g/L)	38.50 ± 4.58	38.51 ± 4.58	38.22 ± 4.66	0.615
GLB (g/L)	33.59 ± 6.79	33.50 ± 6.72	36.47 ± 8.34	<0.001
CD4/CD8	1.77 ± 1.00	1.77 ± 1.01	1.49 ± 0.73	0.025
PLR	199.73 ± 107.62	200.44 ± 108.32	177.92 ± 80.61	0.094
NLR	3.91 ± 2.87	3.86 ± 2.82	5.52 ± 3.81	<0.001
LMR	2.57 ± 1.81	2.58 ± 1.83	2.32 ± 1.36	0.255

*Expressed as the median (M) and interquartile range (IQR).

### Characteristics of the training and validation sets

A total of 2103 patients were randomly allocated to either the training or validation set at a ratio of 7:3, resulting in 1473 patients in the training set and 630 patients in the validation set. Baseline characteristics were comparable between the training and validation cohorts, with no statistically significant differences observed (**Supplementary Table 1**), confirming the effectiveness of the randomization procedure.

### Independent risk factors for IMCs

Univariate and stepwise multivariate logistic regression analyses were performed on the training set to identify risk factors associated with IMC development in lung cancer patients receiving ICI therapy (**Table 2**). Stepwise multivariate logistic regression analysis revealed that female sex and small cell lung cancer (SCLC) were significant risk factors for developing IMCs. Compared with male patients and those with NSCLC, female patients presented a 179% increased likelihood of IMC, whereas those with SCLC presented a 148% increased likelihood. Furthermore, each 1 unit increase in β2-MG, GLB, or the NLR was associated with an elevated risk of IMC, albeit to varying degrees. Conversely, the WBC count, CD4/CD8 ratio, and PLR emerged as protective factors against IMCs. Each unit increase in these variables corresponded with a 3.3%, 5.7%, and 1% reduction in the likelihood of developing IMC, respectively.

**Table 2:** Logistic regression analysis of the risk factors for IMC in the training set.

Variable	No-IMC	IMC	OR (univariable)	OR (multivariable)
Age (Year)	61.79 ± 8.70	60.82 ± 8.10	0.99 (0.95-1.02, *P*=0.464)	
Gender (%)				
Male	1210 (84.67)	29 (65.91)		
Female	219 (15.33)	15 (34.09)	2.86 (1.51-5.42, P=0.001)	2.79 (1.34-5.83, P=0.006)
BMI (%)				
18.5-23.9	830 (58.08)	30 (68.18)		
≥24	500 (34.99)	12 (27.27)	0.66 (0.34-1.31, P=0.237)	
<18.5	99 (6.93)	2 (4.55)	0.56 (0.13-2.37, P=0.430)	
Hypertension (%)				
NO	1107 (77.47)	33 (75.00)		
YES	322 (22.53)	11 (25.00)	1.15 (0.57-2.29, P=0.700)	
Diabetes (%)				
NO	1204 (84.25)	39 (88.64)		
YES	225 (15.75)	5 (11.36)	0.69 (0.27-1.76, P=0.433)	
Pathology (%)				
NSCLC	1124 (78.66)	29 (65.91)		
SCLC	305 (21.34)	15 (34.09)	1.91 (1.01-3.60, P=0.047)	2.48 (1.22-5.06, P=0.012)
Stage (%)				
I-II	50 (3.50)	1 (2.27)		
III	370 (25.89)	7 (15.91)	0.95 (0.11-7.85, P=0.959)	
IV	1009 (70.61)	36 (81.82)	1.78 (0.24-13.27, P=0.572)	
WBC (10^9^/L)	6.96 ± 2.95	6.15 ± 2.34	0.89 (0.78-1.01, P=0.067)	0.67 (0.57-0.80, P<0.001)
Hb (g/L)	124.66 ± 17.78	120.82 ± 19.83	0.99 (0.97-1.00, P=0.159)	
LDH (U/L) *	199.00 [171.00, 240.00]	195.90 [168.00, 240.88]	1.00 (0.99-1.00, P=0.102)	
β2-MG (mg/L)	2.80 ± 1.01	3.25 ± 1.81	1.34 (1.09-1.65, P=0.006)	1.45 (1.12-1.88, P=0.005)
Ca (mmol/L)	2.29 ± 0.15	2.26 ± 0.14	0.25 (0.03-2.12, P=0.203)	
T-cell (%)	942.17 ± 414.97	867.64 ± 311.33	1.00 (0.99-1.00, P=0.238)	
B-cell (%)*	107.00 [60.00, 181.00]	82.50 [55.75, 143.00]	1.00 (0.99-1.00, P=0.154)	
NK (%)	267.27 ± 176.15	260.57 ± 204.62	1.00 (0.99-1.00, P=0.804)	
ALB (g/L)	38.50 ± 4.63	38.26 ± 4.29	0.99 (0.93-1.06, P=0.729)	
GLB (g/L)	33.39 ± 6.80	35.55 ± 6.43	1.04 (1.00-1.08, P=0.038)	1.10 (1.05-1.15, P<0.001)
CD4/CD8	1.78 ± 1.02	1.32 ± 0.48	0.48 (0.30-0.76, P=0.002)	0.43 (0.25-0.73, P=0.002)
PLR	199.58 ± 106.51	169.06 ± 79.78	1.00 (0.99-1.00, P=0.057)	0.99 (0.98-0.99, P<0.001)
NLR	3.83 ± 2.67	4.50 ± 3.36	1.07 (0.98-1.17, P=0.107)	1.60 (1.38-1.87, P<0.001)
LMR	2.61 ± 1.97	2.59 ± 1.47	0.99 (0.85-1.17, P=0.945)	

*Expressed as the median (M) and interquartile range (IQR).

### Establishment and evaluation of the nomogram model

**Figure 2:**
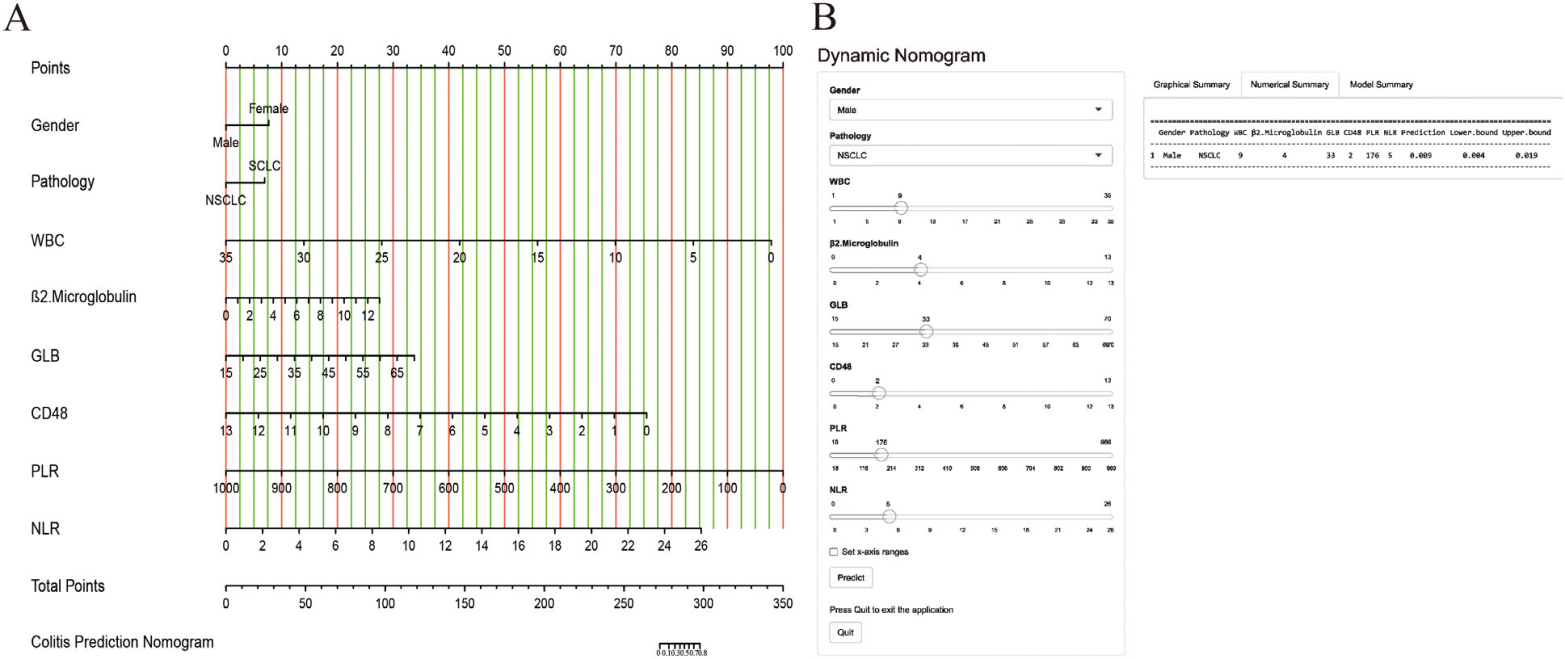
The nomogram model was constructed on the basis of the independent risk factors. **(A)** the nomogram model; **(B)** the online calculator.

A nomogram prediction model, presented in **Figure 2**, was developed on the basis of the results of stepwise multivariate logistic regression analysis to estimate the risk of IMC development in patients with lung cancer receiving ICI therapy. To use the nomogram, points are assigned to each patient on the basis of the scale corresponding to their individual risk factor values. These points are then summed to obtain a total score, which is subsequently mapped to the probability of IMC development.

Furthermore, a user-friendly online calculator has been developed on the basis of a nomogram model to facilitate rapid IMC risk estimation for patients with lung cancer receiving ICI therapy. The calculator is freely accessible at “https://cuch.shinyapps.io/lungcancer_ICIs/”. To increase the clinical utility of the nomogram model, a user-friendly online calculator was developed to facilitate rapid and accessible IMC risk estimation for patients with lung cancer receiving ICI therapy. The calculator allows clinicians and patients to input individual patient characteristics and obtain a personalized risk score. For example, a male patient with NSCLC receiving ICI therapy who presented with a WBC = 9, β2-MG = 4, GLB = 33, CD4/CD8 ratio = 2, PLR = 176, and NLR = 5 would have an estimated IMC risk of 0.009 (95% CI: 0.004–0.019).

**Figure 3:**
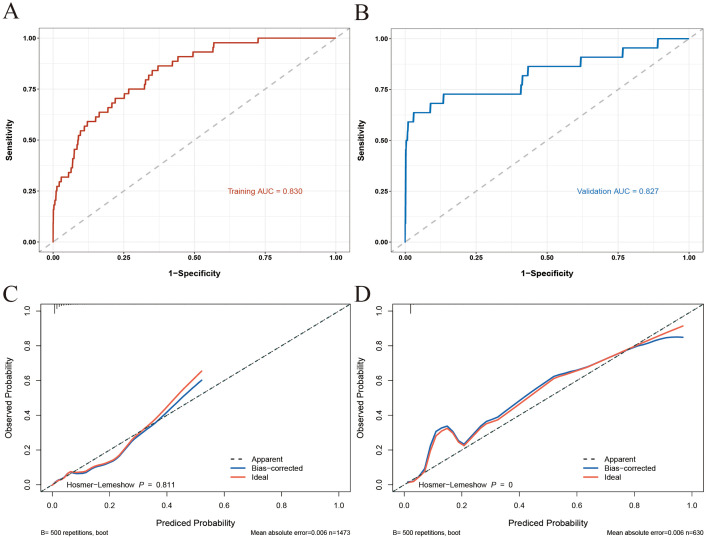
ROC and calibration curves of the nomogram model [**(A)** ROC for the training set; **(B)** ROC for the training set; **(C)** calibration curve for the training set; **(D)** calibration curve for the validation set].

The performance of the nomogram model in discriminating between patients who developed IMC and those who did not was excellent, as evidenced by the AUC values of 0.830 (95% CI: 0.774–0.887) and 0.827 (95% CI: 0.709–0.944) in the training and validation sets, respectively (**Figures 3A, B**). These findings indicate that the model generalizes well and can effectively identify patients at risk of developing IMCs following ICI therapy. The calibration curves for both the training and validation cohorts demonstrated excellent agreement between the predicted and observed probabilities of the IMC, closely aligning with the ideal diagonal line (**Figures 3C, D**). This close alignment indicates good calibration and predictive accuracy.

**Figure 4:**
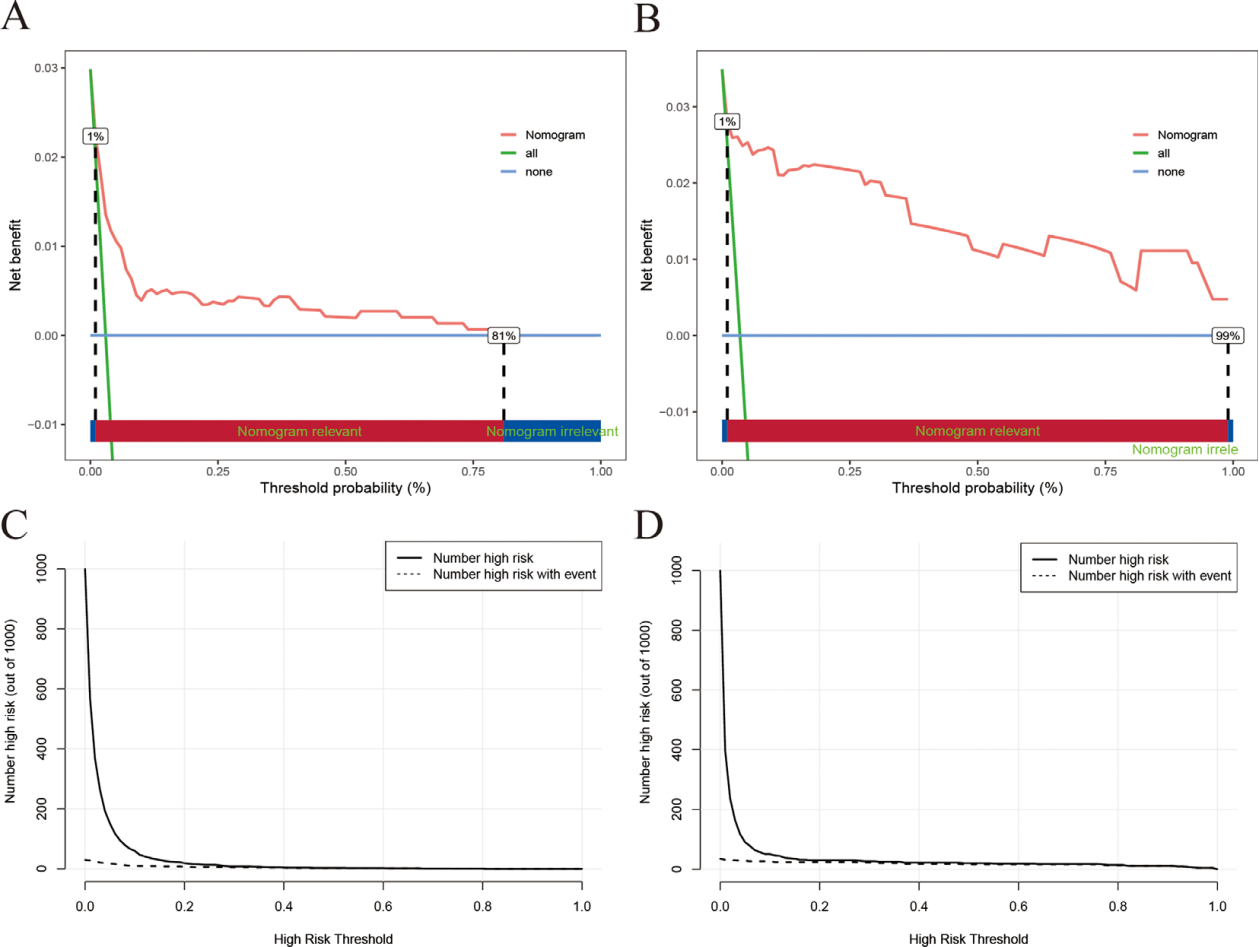
DCA and CIC of the nomogram model [**(A)** DCA for the training set; **(B)** DCA for the training set; **(C)** CIC for the training set; **(D)** CIC for the validation set].

The clinical utility of the nomogram model was further evaluated via DCA and CIC analyses. As illustrated in **Figures 4A, B**, the DCA curves demonstrated that the nomogram model provided greater net benefit than both the “treat-all” and “treat-none” strategies across a wide range of threshold probabilities (1% to 81% in the training set and 1% to 99% in the validation set). This finding suggests that the use of a nomogram to guide treatment decisions for IMC could lead to improved patient outcomes compared with the implementation of a “treat-all” or “treat-none” approach. Furthermore, the CIC analysis corroborated these findings, confirming the potential practical value of the nomogram in real-world clinical settings (**Figures 4C, D**).

## Discussion

Cancer has emerged as the second leading cause of mortality worldwide. Concurrently, ICIs have revolutionized anticancer therapy, significantly improving survival rates in patients with lung cancer (19). However, the expanding indications and increased utilization of ICIs have been accompanied by a substantial increase in the incidence of immune-related adverse events (irAEs), with immune-mediated colitis (IMC) emerging as a prominent concern (20, 21). Identifying the risk factors associated with IMC and developing accurate risk prediction models are crucial steps toward optimizing treatment strategies and improving patient outcomes (22). This study integrated data on tumor characteristics, sociodemographic factors, and relevant hematological indices from a large cohort of patients with lung cancer receiving ICI therapy. This comprehensive analysis aimed to identify independent risk factors for IMC and develop a novel risk prediction model. The developed nomogram model demonstrated excellent discrimination and calibration, suggesting its potential for the rapid and accurate identification of patients at high risk of developing IMC. Importantly, this model relies solely on readily available clinical variables, eliminating the need for invasive procedures such as endoscopic or histopathological examinations. The simplicity, interpretability, and practicality of this model underscore its potential utility in routine clinical practice, facilitating personalized treatment decisions and potentially enhancing the safety of ICI therapy. Additionally, we have developed an online calculator based on the Nomogram model, which is more tailored to the practical circumstances in clinical practice. Clinicians can use the calculation results to provide personalized treatment options for patients, thereby minimizing their IMC risk.

Nomograms have gained increasing popularity in clinical practice because of their ability to transform complex regression equations into intuitive graphical representations, enhancing the interpretability of prediction models. For example, Wang et al. developed a nomogram based on readily available clinicopathological parameters to accurately predict lymph node metastasis in patients with endometrial cancer (23). Similarly, Zhang et al. constructed a user-friendly and cost-effective nomogram utilizing sociodemographic characteristics and routine blood parameters for the early prediction of gestational diabetes mellitus risk with promising accuracy (24). Our study builds upon these advancements by developing a nomogram model for predicting IMC risk in lung cancer patients receiving ICI therapy. On the basis of a multivariate logistic regression analysis, the nomogram integrates multiple predictors via scaled line segments plotted on a single plane, visually representing the interplay between these variables. This user-friendly tool empowers clinicians to rapidly estimate IMC risk in their patients. Furthermore, our nomogram demonstrated excellent discrimination, calibration, and clinical utility in internal validation, suggesting its potential to significantly improve upon traditional diagnostic approaches for IMC. By providing a more accessible and practical tool for clinicians and patients alike, this model has the potential to significantly enhance the management of this important ICI-related complication.

The mechanisms underlying the observed sex disparity in immune-mediated diseases remain an active area of scientific inquiry. Previous studies have revealed significant sex differences in immune responses, with females exhibiting greater susceptibility to certain conditions, including rheumatoid arthritis (RA) and inflammatory bowel disease (IBD) (25). Compared with their male counterparts, women with IBD experience greater X chromosome-related genetic predispositions, a more pronounced hormonal influence on disease activity, and diminished quality of life (26). Recent research has implicated sex-specific variations in the gut microbiome as a contributing factor to the increased incidence of various immune-mediated diseases, including IBD, in females (27). These findings suggest that the gut microbiome may play a role in the observed sex disparity. This heightened immune dysregulation in females may extend to IMCs, as evidenced by the present study’s finding of a 2.79-fold increased risk in women compared with men, a finding that is consistent with previous research (28). Furthermore, the present study revealed a significant association between SCLC and a 2.48-fold increased risk of developing IMC. In clinical practice, PD-L1 inhibitors are the preferred ICI regimen for SCLC, whereas PD-L1 inhibitors are more commonly used in NSCLC (29). Importantly, prior studies have demonstrated that the incidence of IMC varies depending on the specific ICI used (30). One review highlighted that patients receiving PD-L1 inhibitors exhibited a greater likelihood of diarrhea and other gastrointestinal adverse events than did those receiving PD-1 inhibitors (31). The heightened risk of IMC observed in patients with SCLC underscores the critical importance of tailored monitoring and preventive strategies within this patient population.

β2-MG, a low-molecular-weight protein released by activated T and B lymphocytes, has shown diagnostic and discriminatory value in immune-mediated diseases and serves as a potential marker of disease activity in IBD (32). Elevated β2-MG levels are widely recognized as risk factors for various immune-mediated diseases, including IMC (33, 34). Our findings align with these studies, highlighting a positive association between elevated β2-MG and increased IMC risk. Similarly, GLB, an important immune-related marker, plays a significant role in various immune-mediated diseases, including IBD. Previous research has established the GLB as a valuable prognostic biomarker for IBD, accurately reflecting disease severity and progression (35). Given the pathological similarities between IBD and IMC, we hypothesized that the GLB might also have predictive value for IMC risk. Our results support this notion, demonstrating a positive correlation between elevated GLB levels and increased IMC risk. The NLR, another potential predictor identified in our study, has been previously investigated for its diagnostic value in IBD. Marchesi et al. conducted a retrospective study and demonstrated that the NLR can serve as an independent predictor of IBD diagnosis (OR=1.80, *P*=0.016) (36). Similarly, in the context of IMC, an elevated baseline NLR has been significantly associated with IMC development and severity (2), which aligns with our findings.

This study identified several potential protective factors against IMC, including the WBC count, the CD4/CD8 ratio, and the PLR, which have not been extensively reported in previous studies. Leukocytes, essential components of the innate and adaptive immune systems, play crucial roles in combating infections and diseases. While elevated WBC counts are frequently observed in patients with IMC as a marker of systemic inflammation (37), the present findings suggest that higher WBC counts at baseline might be associated with a reduced risk of developing IMC. This potential protective effect could reflect a more robust baseline immune response capable of mitigating the inflammatory cascade associated with IMC development. The relationship between the CD4/CD8 ratio and the IMC remains less clear. Alterations in CD4/CD8 ratios have been linked to immune dysregulation (38) and potentially reflect the prognosis of IMCs (31). However, further large-scale prospective studies are needed to elucidate the complex and interconnected roles of the CD4/CD8 ratio in the context of IMC. PLR, an emerging inflammatory marker, has been associated with various disease states. Previous studies have suggested that PLR fluctuations can aid in assessing treatment response in IMCs (39). Additionally, Poenariu et al. demonstrated the predictive value of the PLR in ulcerative colitis (40), highlighting its potential as a biomarker in inflammatory bowel diseases. Our finding that a high PLR is associated with a reduced risk of IMC warrants further investigation to elucidate the underlying mechanisms involved.

This study has several strengths. First, it represents a large-scale investigation, the first of its kind in Southwest China, to explore risk factors and develop a prediction model for IMCs in lung cancer patients treated with ICIs. Our findings provide valuable insights and a framework for future research in this area. Second, the implementation of rigorous inclusion and exclusion criteria minimized potential biases arising from data heterogeneity.

However, it is important to acknowledge several limitations inherent to this study design. First, the single-center design of this study limits the generalizability and robustness of the prediction model because of the absence of external validation. Future multicenter studies are warranted to validate and refine the developed prediction model. Second, the model did not incorporate endoscopic findings, histopathological data from gastrointestinal biopsies, or genetic information. The inclusion of these variables could provide a more comprehensive assessment of patient characteristics and potentially enhance the model’s predictive performance. Future iterations of the model should prioritize the incorporation of these variables. Finally, the inherent limitations of retrospective studies, such as recall bias and information bias, cannot be completely mitigated. In the future, we will design a prospective cohort study to address these inherent limitations and validate the clinical applicability of the model.

## Conclusion

This study identified eight independent risk factors associated with IMC development in patients with lung cancer receiving ICI therapy. We developed and validated a nomogram prediction model for IMC risk stratification on the basis of these factors. The nomogram model demonstrated excellent discrimination, calibration, and clinical utility, suggesting its potential as a readily applicable tool for the rapid and accurate assessment of IMC risk in routine clinical practice. The identification of high-risk individuals via this model could facilitate personalized treatment decisions, potentially enabling timely interventions to mitigate IMC development and enhance the safety profile of ICI therapy in this patient population.

## Data Availability

The raw data supporting the conclusions of this article will be made available by the authors, without undue reservation.
