# Supplementary material for: Development and validation of a nomogram for predicting immune-mediated colitis in lung cancer patients treated with immune checkpoint inhibitors: a retrospective cohort study in China
Source: Front Immunol. 2025 Jan 30;16:1510053. doi: 10.3389/fimmu.2025.1510053 (PMC11821966; doi:10.3389/fimmu.2025.1510053)
Supplement: Supplementary file 1 [file Table1.docx]

Supplement Table 1 Characteristics of the training and validation sets.

| Variable | Training set(n=1473) | Validation set(n=630) | *P* |
| --- | --- | --- | --- |
| Age (Year) | 61.76±8.69 | 61.46±9.31 | 0.469 |
| Gender (%) |  |  | 0.575 |
| Male | 1239 (84.11) | 523(83.02) |  |
| Female | 234(15.89) | 107(16.98) |  |
| BMI (%) |  |  | 0.320 |
| 18.5-23.9 | 860(58.38) | 356(56.51) |  |
| ≥24 | 512(34.76) | 238(37.78) |  |
| <18.5 | 101(6.86) | 36(5.71) |  |
| Hypertension (%) |  |  | 0.955 |
| NO | 1140(77.39) | 489(77.62) |  |
| YES | 333(22.61) | 141(22.38) |  |
| Diabetes (%) |  |  | 0.201 |
| NO | 1243(84.39) | 546(86.67) |  |
| YES | 230(15.61) | 84(13.33) |  |
| Pathology (%) |  |  | 0.321 |
| NSCLC | 1153(78.28) | 506(80.32) |  |
| SCLC | 320(21.72) | 124(19.68) |  |
| Stage (%) |  |  | 0.126 |
| I-II | 51(3.46) | 19(3.02) |  |
| III | 377(25.59) | 188(29.84) |  |
| IV | 1045(70.94) | 423(67.14) |  |
| WBC (${10}^{9}$/L) | 6.93±2.93 | 6.87±2.79 | 0.657 |
| Hb (g/L) | 124.55±17.85 | 124.05±17.60 | 0.554 |
| LDH (U/L) * | 198.00 [171.00, 240.00] | 203.00 [172.00, 251.75] | 0.073 |
| β2-MG (mg/L) | 2.82±1.04 | 2.94±1.11 | 0.077 |
| Ca (mmol/L) | 2.29±0.15 | 2.29±0.16 | 0.485 |
| T cell (%) | 939.94±412.37 | 985.41±432.25 | 0.023 |
| B cell (%) * | 107.00 [60.00, 180.00] | 106.00 [59.00, 173.00] | 0.581 |
| NK (%) | 267.07±176.99 | 256.03±178.74 | 0.192 |
| ALB (g/L) | 38.49±4.62 | 38.52±4.50 | 0.904 |
| GLB (g/L) | 33.45±6.79 | 33.92±6.79 | 0.144 |
| CD4/CD8 | 1.77±1.01 | 1.76±0.99 | 0.820 |
| PLR | 198.67±105.92 | 202.22±111.54 | 0.489 |
| NLR | 3.85±2.70 | 4.04±3.23 | 0.163 |
| LMR | 2.61±1.96 | 2.49±1.42 | 0.163 |

Note：*Expressed as median (M) and interquartile range (IQR).
